# 

**Published:** 1880-07

**Authors:** 


ESTABLSIHED IN 1855.

Volume XVIII. **JULY, 1880.** Number 4

### ATLANTA MEDICAL AND SURGICAL JOURNAL.

*MONTHLY:, THREE DOLLARS A YEAR, IN ADVANCE.*

EDITOR:

J. G. WESTMORELAND, M.D.,

*Professor of Materia Medica in Atlanta Medical College.*

H. H. DICKSON, PUBLISHER.

*PAX ET SCIENTIA, SED VERITAS SINE TIMORE.*

ATLANTA, GEORGIA:,

*H. H. DICKSON, BOOK AND JOB PRINTER AND BOOKBINDER.*

1880.

